# Food Habits, Lifestyle Factors, and Risk of Prostate Cancer in Central Argentina: A Case Control Study Involving Self-Motivated Health Behavior Modifications after Diagnosis

**DOI:** 10.3390/nu8070419

**Published:** 2016-07-09

**Authors:** Sandaly O. S. Pacheco, Fabio J. Pacheco, Gimena M. J. Zapata, Julieta M. E. Garcia, Carlos A. Previale, Héctor E. Cura, Winston J. Craig

**Affiliations:** 1 Center for Health Sciences Research, School of Medicine & Health Sciences, Universidad Adventista del Plata, Libertador San Martín, 25 de Mayo 99, Entre Ríos 3103, Argentina; sandalyoliveira@doc.uap.edu.ar (S.O.S.P.); gimena_zapata@hotmail.com (G.M.J.Z.); emilianagarcia@hotmail.es (J.M.E.G.); carlos.previale@sanatorioadventista.com.ar (C.A.P.); 2 Institute for Food Science and Nutrition, Universidad Adventista del Plata, Libertador San Martín, 25 de Mayo 99, Entre Ríos 3103, Argentina; wcraig@andrews.edu; 3 Division of Clinical Oncology, Sanatorio Adventista del Plata, Libertador San Martín, 25 de Mayo 255, Entre Ríos 3103, Argentina; 4 Division of Radiation Oncology, Unidad de Terapia Radiante Entre Ríos, Paraná, Além 654, Entre Ríos 3100, Argentina; edcura@yahoo.com.ar; 5 Department of Public Health, Nutrition and Wellness, School of Health Professions, Andrews University, Berrien Springs, MI 49104, USA

**Keywords:** food habits, lifestyle factors, prostate cancer, fruits and vegetables, health behavior modification, Argentinean men

## Abstract

Cancer is the second most important non-communicable disease worldwide and disproportionately impacts low- to middle-income countries. Diet in combination with other lifestyle habits seems to modify the risk for some cancers but little is known about South Americans. Food habits of Argentinean men pre- and post-diagnosis of prostate cancer (*n* = 326) were assessed along with other lifestyle factors. We studied whether any of the behaviors and risk factors for prostate cancer were found in men with other cancers (*n* = 394), compared with control subjects (*n* = 629). Before diagnosis, both cases reported a greater mean consumption of meats and fats and lower intakes of fruits, green vegetables, cruciferous vegetables, legumes, nuts, seeds, and whole grains than the controls (all *p* < 0.001). After diagnosis, cases significantly reduced the intake of meats and fats, and reported other dietary modifications with increased consumption of fish, fruits (including red fruits in prostate cancer), cruciferous vegetables, legumes, nuts, and black tea (all *p* < 0.001). Additional lifestyle aspects significantly predominant in cases included a reduced quality of sleep, emotional stress, low physical activity, tobacco smoking, alcohol consumption, living in rural areas, and being exposed to environmental contaminants. Argentinian men were predisposed to modify their unhealthy dietary habits and other lifestyle factors after cancer diagnosis.

## 1. Introduction

Global prevalence of non-communicable diseases (NCDs) is one of the main concerns of health professionals worldwide. According to the World Health Organization (WHO), cardiovascular diseases, cancers, respiratory diseases, and diabetes accounted for 82% of all NCD deaths in 2015 [1]. The number of NCD deaths attributed exclusively to cancers is almost 50% higher than the total number of NCD deaths due to respiratory diseases and diabetes together. Despite the fact that each of these diseases is characterized by complex pathophysiologic mechanisms, similar health strategies have been proposed for the prevention of many NCDs. This is particularly true for cardiovascular diseases, respiratory diseases, and type 2 diabetes but strategies focusing on cancer prevention are currently an area of intense investigation [2]. Extrinsic factors such as diet and other nutritional aspects together with lifestyle behaviors have been suggested to be associated with cancer [3]. Thus, identification of risk factors is especially important for establishing specific cancer prevention actions. Results from a 2016 investigation by Wu et al. proposed that extrinsic factors contribute to two thirds or more of most common cancers [4]. The prevalence of prostate and colorectal cancers, for instance, are among the highest in highly developed countries and incidences vary up to 10-fold with greater disease burden in economically transitioning countries [5,6]. Specific food habits, lifestyle factors, and environmental components may be responsible for differences in worldwide cancer distribution. The first population-attributable fraction study on cancer risk in a South American country estimates that 46% of the expected deaths in men at 2020 will occur from preventable causes, which includes a suboptimal diet with low consumption of fruits and vegetables [7].

Prostate cancer is the second most prevalent cancer among men in the world with high incidence but relatively low mortality rates compared with lung and colorectal cancers [8]. Besides the small number of known risk factors for prostate cancer such as family history, age, and race, recent investigations have proposed that there might be other lifestyle factors associated with diet, physical activity, and sleep that protect from or predispose to prostate cancer development. Yang et al. (2015) recently demonstrated that unhealthy dietary patterns increased prostate cancer -specific and total mortality [9], while high intakes of plant-based meals, lycopene-rich foods, and whole grains seem to offer substantial protection against prostate cancer [10,11]. Adequate levels of physical activity were also lately associated with increased survival after prostate cancer diagnosis [12], while insufficient sleep was associated with advanced prostate cancer risk [13]. Single factors may influence cancer prevention or development but the wider impact of combined lifestyle factors is a matter of recent debate. There are new studies that assessed the effect of multiple lifestyle practices on cancer risk and diet is one of the key factors [14,15,16]. Nevertheless, most of these studies were conducted in well-developed countries where food habits and other lifestyle factors may be influenced by common socioeconomic and cultural characteristics. To date, very little research considering food habits and multiple lifestyle factors has been done in South America where several health disparities coexist and NCDs are disproportionately abundant [17,18]. Almost three quarters of NCD deaths occur in people from low- to middle-income countries [1], and the worldwide number of health interventions targeting multiple risk factors was recently summarized with only 0.5% of all research being done in South America [19]. Therefore, we aimed in this study to identify the existence of multiple health behaviors including food habits and lifestyle-related factors reported by Argentinean men diagnosed with prostate cancer. We were also interested in assessing those factors after the prostate cancer diagnosis, since prostate cancer mortality within five years is relatively low and recent evidence indicates that healthy lifestyle modifications post-diagnosis may positively impact quality of life and survival [9,16,20,21]. We also looked at whether some of these health behaviors and risk factors for prostate cancer would be found in men with other types of cancers, to see if common factors exist.

## 2. Methods

### 2.1. Study Population

Men were invited to participate in this study during appointments to hospitals and medical centers in the provinces of Santa Fe and Entre Ríos, central areas of Argentina, between 2011 and 2013. The cases, consisting of two different groups, were under treatment of a histopathologically confirmed prostate cancer (*n* = 326) or other cancer (*n* = 394) (primarily lung and colorectal cancer). Men of a similar age and residing in the same province as the cases and who did not receive a cancer diagnosis were invited to participate as controls (*n* = 629) after their medical visit. One hundred seventy-nine participants in each group was calculated as the minimum sample size required to measure a medium effect size with a 90% power at α = 0.05.

The study protocol was reviewed and approved by the Research Review Board (registered under the #42.2011) and Ethics Committee of the River Plate Adventist University (resolution #2-2012-05.1/2012), and includes patient consent after proper explanation of the study.

A structured questionnaire was used for targeting potential protectors and main lifestyle related-risk factors for NCDs, especially those associated with male cancers [22,23,24]. The questionnaire had two main sections with questions on: (1) sociodemographics and general health information; (2) food habits and lifestyle-related factors.

### 2.2. Sociodemographics and Health Information

Participants provided information on age (years), education (elementary/secondary school, postsecondary and university), actual marital status (singled, married, widowed, and divorced), main labor activity (rural, industrial, commercial, autonomous professional, and other), and life residency (city or rural with less than 2500 people grouped, as characterized in Argentina). Body Mass Index (BMI, kg/m^2^) was calculated from reported weight and height. Data collected also included the initial reason for seeking medical assistance that finally led to a cancer diagnosis (regular health check-up or presence of a given symptom), family history of cancer (any type of cancer in relatives, including grandfather/grandmother and participant’s siblings), and history of a medical diagnosis of prostatitis, urinary tract infections, arterial hypertension, diabetes, thyroid dysfunction, depressive disorder, and correlated medications. We also inquired about life-disturbing events and important emotional stressors in the last years prior to cancer diagnosis, such as those related to divorce, breakup of a significant relationship, serious illness or death in close family members, loss of a long-time job, and other personal issues that negatively impacted life experiences.

### 2.3. Food Habits and Lifestyle-Related Factors

Questions in this section were divided in two parts concerning present (controls and cases) and past practices and life experiences before cancer diagnosis (cases). We clarified, firstly, that participants should answer the questions based on their most common and typical habit that best represents their lifestyle, whether in the preceding years before cancer detection or current behaviors. Regarding diet, men were asked about their regular consumption of common foods and food groups usually found in Argentinean local markets: meat, fish, milk and milk products, fruit (pears, apples, peaches, grapes, bananas, oranges, mandarins, lemons, etc.), red fruits (strawberries, cherries, tomatoes, watermelons, red grapefruit), green salads (lettuce varieties, arugula, escarole, spinach, watercress, etc.), cruciferous vegetables (cabbage, broccoli, cauliflower), legumes (lentils, black beans, garbanzos, peas, soy), seeds (sunflower, chia, flax, sesame, etc.), nuts (walnuts, almonds, peanuts, etc.), whole grain cereals (oats, wheat, rice, etc.), sugary foods including sweet beverages and confectionary, foods with high contents of trans and saturated fats including fries, factura (a kind of croissant regularly eaten in Argentina), and fatty desserts, coffee, black tea, and mate. For these items, examiners specified an approximate regular portion of each element using a non-quantitative food frequency questionnaire (FFQ), and participants reported the habitual frequency using the following categories: (1) never; (2) 1–2 times/week; (3) 3–4 times/week; (4) 5–7 times/week. Participants also reported the usual water consumption (cups per day).

For other lifestyle factors, participants were asked about: physical activity (30 min or more of moderate aerobic activity at least 3 or more times/week on a regular basis); sleep pattern and usual evening sleep duration in hours (6 or fewer, between 6 and 8, and more than 8), sleep interruptions (more than two times per night), difficulty sleeping and the use of medications for sleeping; tobacco use (present, past, and passive history of smoking); and alcohol consumption (daily, occasional, or never). Participants were also asked about their contact with agricultural chemicals (indirect exposure for those living in areas less than 1000 m of fumigation zones and direct exposure for those who handle, apply, or sell agrochemicals) [25], time of exposure (years), identification of substances by commercial or chemical names (grouped according to class of pesticides), and personal protective garments while using agrochemicals [26]; and general exposure, to compounds possibly associated with prostate cancer such as cadmium, metallic dust, and liquid fuel combustion [27].

We also applied a descriptive analysis considering seven unhealthy habits, combining five food habits with two other lifestyle-related factors, in order to determine their accumulation pattern in cases and controls. The following factors were considered risk behaviors: intake of fresh fruits, red fruits, and green salads was <3 times/week, for each group of food [28]; the intake of meats and fatty foods was three or more times/week, for each group of food [3]; physical activity was <3 times/week; and current tobacco smoking [7].

### 2.4. Statistical Analyses

Descriptive analyses were carried out for sociodemographic and health information, and food habits and lifestyle-related factors for cases and controls. Chi-square Itest was used for assessing differences in categorical variables among participants of the three groups. McNemar’s test was applied for examining the significance of the differences between pre- and post-diagnosis habits for individual variables inside each group, with reference to prostate cancer cases and other cases separately. In order to calculate odds ratios (ORs) and 95% confidence intervals we used multiple logistic regression models, adjusted for age, level of education, marital status, BMI, and family history of cancer. For that we compared dietary habits, place of residency (urban or rural), physical activity (three or more times a week), history of important emotional distress, and use of statins. For tobacco smoking we considered the categories active, passive, or past smoking. SPSS Inc., (Chicago, IL, USA) version 17 software was used for statistical analyses. *P* values < 0.05 were considered statistically significant.

## 3. Results

Men in both cancer cases had, on average, a small but significantly higher age than controls, lower body mass index (BMI) and, particularly for men in the prostate cancer group, a lower level of education. Prostate cancer was detected mainly during medical checkups, while the majority of men with other types of cancer only looked for a physician after the onset of symptoms. In both cases the overall history of cancers in the family, considering near or distant relatives, was greater than controls. Medical history of prostatitis and arterial hypertension was more common in men with prostate cancer while other cancer cases also showed a higher prevalence of arterial hypertension than controls. The use of statins was more prevalent in controls. A major stressor and emotionally life-disturbing event was more prevalent in cases than in controls (Table 1). Although the majority of the studied population was not directly exposed to agrochemicals, cases showed greater exposure, both direct and indirect contact, than the control subjects. Cancer cases had a substantially higher contact with organophosphorus compounds than controls in addition to a more frequent history of inadequate protective measures. Housing in a rural area, defined as fewer than 2500 people, was more prevalent in cases than controls (Table 1).

Regarding diet and food habits, before diagnosis, cases reported a greater mean consumption of meat, fat, and coffee than the controls. They also ate significantly less fruit, vegetables, green salads, cruciferous vegetables, nuts, seeds, and whole grains (Table 2). Conversely, after cancer diagnosis, men significantly reduced the intake of meat, fat, and coffee and reported other diet modifications with increased consumption of fish, fruit, including red fruits (only in prostate cancer cases), cruciferous vegetables, legumes, nuts, and black tea. Similar consumption of sweet beverages, mate, and fish were found before cancer diagnosis in all groups including controls. Daily water intake was significantly lower in prostate cancer cases compared to the remaining participants (Table 2).

Before diagnosis both cases had an overall reduced quality of sleep than controls with a higher consumption of sleep medications, more sleep disruptions, and difficulty falling asleep. After cancer diagnosis these trends were maintained, with a further reduction in hours of night sleeping and an increased use of sleep medications among cases compared with controls (Table 3). Cases were significantly less engaged in regular physical activity than the controls, with reduced activity after diagnosis. Previous to cancer diagnosis, cases consumed more alcohol and used more tobacco than controls. This behavior changed significantly after diagnosis. Tobacco use was more prevalent in men with other types of cancer than in men with prostate cancer. The use of antidepressant medications was similar in all groups but its consumption increased significantly after diagnosis of cancer in both cases (Table 3).

Living in an urban area, participating in physical activities, and eating fruit and vegetables, including red fruits, green salads, and cruciferous vegetables, three or more times a week was inversely associated with a risk of prostate cancer. The same association was observed for the other cancer cases regarding the intakes of fruit, cruciferous vegetables, whole grains, and dairy, and living in an urban area. While the intake of coffee was associated only with prostate cancer risk, the higher intake of foods rich in fat, meat, and black tea was associated with risk of developing prostate cancer and other cancers. Familial history of cancer and a significant life stressor were also associated with risk in both cases. In this study, the usage of tobacco was associated with a risk for other cancers but not for prostate cancer (Table 4).

An integrative analysis of seven risky health behaviors (low intake of fresh fruit, red fruits, and green salads; high intake of meat and fatty foods; insufficient physical activity; and tobacco usage) revealed a directly proportional association between the number of risk factors accumulated and the presence of a cancer diagnosis. Before diagnosis, both cancer groups had a higher number of risky health behaviors with 78.1% of the prostate cancer cases and 80.5% of the other cancer cases accumulating in their lifestyle 4–6 risky behaviors, whereas 83.4% of the control subjects accumulating 1–4 risky factors. After cancer diagnosis, cases changed their behaviors and prostate cancer cases moved towards the distribution pattern found in fewer risk factors (Table 5).

## 4. Discussion

In this study, cancer cases in both groups presented with a substantially higher number of risk factors related to lower levels of education, familial history of cancer, prostatitis (in prostate cancer cases), and unhealthy lifestyle habits associated with diet, physical activity, sleep, tobacco smoking, alcohol consumption, emotional distress, and exposure to agrochemicals, than the control subjects. After cancer diagnosis, a significant number of cases reported favorable changes in regard to diet, tobacco and alcohol consumption, which may interfere with disease progression and benefit quality of life if these healthier behaviors are sustained. These findings add to the growing literature suggesting that unhealthy lifestyle behaviors may be important factors influencing cancer development. Among all the participants of this study, prostate cancer cases had the lower educational level, which may be associated with unhealthy choices [29]. However, most of the prostate cancer diagnoses were initiated during medical check-ups, which suggests a positive impact of public health campaigns for prostate cancer screening. Similarly, Bekker-Grob et al. (2013) found that men from the southwest of the Netherlands with lower educational levels decided more easily on a prostate cancer screening [30]. In contrast with the diagnosis of prostate cancer, the majority of men with other cancers only searched for medical aid after onset of symptoms. As suggested by the study of Okada et al. (2016) in Chile, early detection approaches and cancer prevention policies in South America should be reconsidered, especially for the most prevalent cancers in men such as colorectal and lung cancer [31]. According to the National Association for Cancer Registry in Argentina (2011), prostate, lung, colorectal, stomach, and bladder cancers are among the highest prevalent cancers in Argentinean men [32].

The increased risk of cancer in first generations from relatives with prostate cancer has been extensively studied but the latest investigations showed a larger effect including second and third degree relatives influencing risk of prostate cancer, with equivalent risks from history of prostate cancer from the paternal and maternal sides [33]. Segregation analysis indicates that prostate cancer resembles adult-onset family cancer syndromes with the participation of several genes [34]. Pakkanen et al. (2007) showed that Mendelian recessive inheritance is also associated with prostate cancer and paternal regressive coefficient values, influenced by lifestyle choices and environmental factors, modulates prostate cancer risk [35]. A recent study found that prostate cancer was 25 times higher among members of a large Familial Ovarian Cancer Registry [36], highly suggestive of an effect of non-prostate cancers history on prostate cancer risk. With respect to the broad approach we have used for ascertaining history of cancer, inquiring about any type of cancer in close or distant relatives is useful, since we detected a significantly higher familial prevalence of cancer in both cases compared with controls. Nevertheless, these findings warrant further studies since our data were limited. Besides inherited genetic susceptibilities, progenitors may influence their descendants through socialization and impart lifestyle preferences and inclinations, predisposing food choices, physical activity practices, alcohol usage, and tobacco smoking [37,38,39]. Furthermore, regulation of DNA expression through epigenetic mechanisms is influenced by the environment and dietary habits of progenitors and has been suggested to be associated with metabolic syndrome, neurodegenerative diseases, and cancers in their descendants [40,41]. Recent findings propose that lifestyle habits can exert influences in family environments since spouses of long-lived families also enjoy relatively healthy lives [42]. Thus, health intervention strategies to make families aware of the scope of their influence on healthy lifestyle behaviors are required [43].

Prostatitis has been associated with an elevated risk for prostate cancer [44] and loss of tumor suppressor genes in prostate tissues [45]. Interesting, 15% of prostate cancer cases in our study had a history of prostatitis, exceeding the usual prevalence (2.2%–9.7%) [46] and also the prevalence we found in the other cancer cases (3.8%) and controls (10%). Among participants of our study, prostate cancer cases consumed significantly less water. Chronic prostatitis was recently shown to be higher in individuals with low water intake [47]. Very few studies have considered beverage habits around the world, particularly water intake in patients diagnosed with cancer. Therefore, it would be interesting to corroborate and extend our initial observations in further investigations.

When asked about a major life-disturbing event, both cases presented a more prevalent history of emotional distress than controls. Li et al. (2014) found a greater prostate cancer risk in men who experienced important emotional distress [48]. Although there are many attempts trying to link emotions such as worry, anger, fear, anxiety, and depressive feelings with the etiology of malignant tumors, it has been difficult to demonstrate specific biological causal links. Nevertheless, studies have shown that altered emotions and psychological distress caused by persistent life-troubling situations may affect several aspects of the lifestyle such as reducing the hours of rest and sleep [49], modifying diet preferences favoring higher intakes of palatable foods rich in fat and sugar [50], prompting social isolation and intensifying alcohol and tobacco consumption [51], and reducing physical activity levels [52]. All of these lifestyle factors have been shown to affect cancer risk and mortality. Perceived emotional stress and lack of social support, in a study with 4015 Swedish men, accounted for a 66% increased risk of prostate cancer mortality [53]. Thus, comprehensive psychosocial care will assist oncological patients in making healthy lifestyle decisions. Integration of public and private health initiatives for addressing this need is recommended [54].

Suboptimal diet has been shown to influence carcinogenesis and it is one of the most studied lifestyle-related factors associated with NCDs [3,55,56]. The World Cancer Research Fund (WCRF) and the American Institute for Cancer Research (AICR) have been regularly examining new evidence for the impact of diet on cancer and, while tremendous advances have been made [57], it continues to be an area under intense investigation. Epidemiological studies have attempted to specify certain foods that may prevent or favor cancer development but it is challenging to translate this information into the clinical scenario [58]. In this study, we focused on the certain groups of foods frequently mentioned in the NCDs literature, including those targeted at cancer prevention strategies such as fruit, red fruits, legumes, green salads, cruciferous vegetables, and nuts [55,59]. Although the data collection does not thoroughly discriminate all types of foods and precise quantities, it may be useful for assisting clinicians, urologists, oncologists, and other professionals in making health recommendations. The value of nutrition education has been highlighted by other studies in which most of the patients and their partners requested from different health professionals dietary information soon after a prostate cancer diagnosis [21,60,61]. Indeed, the results obtained by using our non-quantitative FFQ were able to find differences between food consumption among cases and controls revealing distinct habits and preferences among these groups. Showing similarity to a “Western diet” pattern [9], cases of our study consumed more fats and meats, and lower intakes of plant foods than controls. Selenium, folate, vitamin C, D, E, K, phytosterols, resveratrol, and other health-promoting phytochemicals are found mainly in a plant-based diet. These substances have been associated with lower incidences of several cancers [62]. These and other nutritional compounds may exert anti-tumor activities, inhibiting proinflammatory transcription factors such as NF-ΚB, diminishing arachidonic acid, inflammatory prostaglandins, angiogenesis, oxidative stress, and potentially chemo- and radio-sensitizing tumor cells [3,63]. Enterolignans are produced by the gut microbiome after intake of dietary fiber and have been suggested to exert important biological functions, preventing chronic diseases such as cancers [64,65]. Similarly, a Mediterranean-style diet or a vegetarian diet characterized by good quantities of fruit, vegetables, complex carbohydrates, healthy fats, and moderate amounts of proteins may help prevent cancer development/progression [16,66,67]. We observed that a diagnosis of cancer prompted many men of our study to make self-motivated positive dietary changes, increasing the intakes of fruit and vegetables and decreasing fat and foods from animal sources. Avery et al. (2013) found that one third of men diagnosed with prostate cancer spontaneously adopted a healthier diet [20], while in another study about one half improved their diet [68]. The adoption of a healthy lifestyle after a cancer diagnosis has been associated with a better quality of life and superior clinical outcomes [9,69]. Intakes of healthy diets reduce biomarkers of oxidative stress in men diagnosed with prostate cancer [70]. Higher mortality after prostate cancer diagnosis was associated with low intake of vegetables and high intake of saturated fats in the recent Physicians’ Health Study (PHS) [71]. Also, the European Prospective Investigation into Cancer and Nutrition (EPIC) study reported a lower risk of some cancers in 47,479 Italians following a Mediterranean diet rich in raw vegetables, fruit, and olive oil [66].

Growing evidence for the adoption of a healthier lifestyle for patients diagnosed with cancer has being fostered by recent studies and the integration of these concepts into oncological care is novel. Research in this area is warranted, particularly in Latin America and the Caribbean, where very few studies have been done and cancer deaths are projected to double from 2012 to 2030 [72]. This is important given the traditional Argentinean diet, high in red meat, saturated fats, and simple carbohydrates and low in vegetables, all of which increase the risk of prostate cancer [73]. We also found that consumption of coffee and black tea was significantly more prevalent in cases than in controls and logistic regression analysis showed a risk associated with these beverages. After cancer diagnosis, both cases decreased coffee intake but increased black tea consumption. Recent epidemiological studies on coffee did not find an association with a prostate cancer risk [74,75] and some inversely associated coffee intake with prostate cancer risk [76]. However, green tea has been largely associated with higher antioxidant capacity, and a recent clinical trial with prostate cancer patients did find benefits from green tea on several biomarkers but did not show any benefit to black tea consumption [77].

Adequate sleep seems to be crucial for biological regeneration through the activation of cell repair mechanisms mainly by the action of melatonin, which participates in the regulation of hormones, fatty acids metabolism, energy expenditures via insulin/IGF-1, and adult stem cells differentiation/proliferation [78,79]. Investigations indicate that disruption of the circadian rhythm is associated with impaired immunity, activation of pro-inflammatory cytokines [80,81], and cancer development [82,83]. Nevertheless, only a few studies have examined the impact of sleep on prostate cancer and other cancers in men, with some suggesting a causal link [84,85,86] but others suggesting no link [87,88]. A recent prospective study found that men who self-reported sleep problems had significantly lower melatonin levels in urinary samples and this group presented a four-fold increased risk of developing advanced prostate cancer [13]. Shiftwork has been implicated with prostate cancer risk and a strong positive association with Prostatic Specific Antigen (PSA) levels was found [89]. In our study, while we did not find a risk associated with insufficient sleep, there was a statistically significant difference in the descriptive analysis, showing worst sleep parameters in both cancer groups, but more prominently in prostate cancer cases. These changes in sleep alterations could be corroborated, at least in part, by the substantial high use of sleeping medications among men before prostate cancer diagnosis (31.6%) and in men with other cancers (23.6%), in regard to control subjects (15.1%). Petrov et al. (2014) found that 9.6% of their subjects used prescribed sleep medications and 11.1% used over-the-counter sleep aids [90]. Analogous to other investigations, where cancer patients presented sleep disturbances after cancer diagnosis and treatment [91], both cancer groups of our study significantly increased the consumption of sleep medications (up to 44.7% of men with other cancers) and antidepressants (up to three times more than at baseline), possibly associated with cancer-related fatigue and depression cluster conditions [92]. However, management of sleep and depression includes also non-pharmacological approaches. Men diagnosed with or at risk of cancer might benefit from health interventions designed for improving quality of sleep and depression mitigation as shown, for instance, with cognitive brain therapy [93].

Being physically active is an important aspect of a healthy lifestyle and while the benefits for the cardiovascular system have been extensively demonstrated, it is an emerging area of interest in prevention of cancers [94,95]. In the preceding years before diagnosis, most of the cases in our study were not engaged in regular physical activity (PA) and PA levels were even lower after cancer diagnosis. Friedenreich et al. (2016) asked men with prostate cancer diagnosis about their lifetime history of PA and found a direct association for those performing more PA, including recreational activity and non-sedentary occupational activity, with a lower risk of prostate cancer death [12]. In our study we found that three or more times a week of PA was associated with prostate cancer risk reduction. In the clinical setting, however, it is challenging to establish specific recommendations of PA for cancer patients due to a variety of personal conditions and oncologic treatments. Nevertheless, sedentary behaviors should be discouraged, since a growing body of research associates sitting time with all-cause mortality [96], including mortality for some cancers [97,98]. The American Cancer Society recommends 150 min of moderate or 75 min of vigorous PA per week for cancer risk reduction [99]. Just half an hour of daily walking, considered a low-intensity PA, was associated with reduced liver cancer mortality [100], and modest amounts of weekly vigorous PA for 3 h (playing tennis, biking, or swimming) seemed to decrease prostate cancer specific mortality [101] and improved quality of life in prostate cancer patients [102].

Tobacco is recognized as the major preventable risk factor for most cancers [103], contributing to one in three cancers [104]. Tobacco has been associated with lung, oral cavity, pharynx, larynx, esophagus, stomach, liver, urinary tract, cervix, and other cancers [105]. However, the association of tobacco with a prostate cancer risk is not so clear [106]. While in our study the habit of smoking was significantly more prevalent in both cancer groups than in controls, we did not find an increased risk of prostate cancer associated with smoking. Most epidemiological investigations have not found a prostate cancer risk associated with smoking [107,108], while some others did find an association [109]. However, post-diagnosis tobacco exposure is adversely associated with prostate cancer prognosis. Recent studies show greater expression of immune/inflammatory genes in prostate tumor tissues [110] and higher biochemical disease recurrence, metastasis, and mortality in prostate cancer patients who smoke [111,112,113]. In our study, about 15% of men with prostate cancer spontaneously quitted smoking after diagnosis, while 40% of men with other cancers quit, a significant reduction in both cases. Hence, men facing a cancer diagnosis might be susceptible to lifestyle changes, creating an opportunity for a “teachable moment” [114]. Since in our study there was an important difference among cases regarding smoking reduction, we can also consider that the etiology of prostate cancer is not stereotyped with tobacco as might be the case with other cancers.

Along with smoking, alcohol consumption has been an even more controversial subject in the etiology of prostate cancer but not with many other types of cancers [115]. Most research has not found a direct effect of alcohol on prostate cancer development; however, some studies found an increased risk of prostate cancer considering lifetime alcohol consumption [116], and prostate cancer aggressiveness [117]. In our study, both cases were significantly more involved in overall alcohol intake than controls but logistic regression analysis did not show any associated risk. Nevertheless, alcohol consumption may be associated with co-occurring unhealthy habits, constituting an unidentified confounding feature of epidemiological studies. Hansel et al. (2015) demonstrated that wine consumers favored purchasing healthy foods while beer consumers were more likely to buy unhealthy foods [118]. In our study, more than half of men from both cases substantially reduced their everyday consumption of alcohol following diagnosis. Little research has assessed alcohol intake after prostate cancer diagnosis. Hackshaw-McGeagh et al. (2015) reported positive changes with reduction of alcohol intake in approximately one quarter of men diagnosed with prostate cancer [119]. It would be important, therefore, to investigate alcohol effects following prostate cancer diagnosis and its association with concomitant food habits.

Additional factors may be connected with cancers in men such as those resulting from occupational and environmental exposures [120]. Argentina is among the three largest producers of soy, corn, and other crops [121]. Prostate cancer has been found to be associated with the use of some herbicides [122], organophosphate, and organochlorine insecticides [120,123]. However, none of these associations appear to be conclusive. In our study, there was not a significant risk for prostate cancer as well as other cancers associated with pesticide exposures, but we cannot rule out the possibility since prolonged specific type and time, direct and indirect, pesticide exposures were more prevalent in cases than in controls. Also, more than half of the cases directly exposed to pesticides did not use adequate protective measures for handling pesticides, suggesting higher vulnerability to contaminants. We also found a lower risk of prostate cancer and other cancers in men living in urban areas (not necessarily large cities), in contrast with those living in rural areas. However, this may be associated with health services accessibility, larger variety of foods, water treatment, and probably less agrochemical exposures among other factors [124]. Studies show that although the predicted risk of cardiovascular diseases seems to be lower in rural than in urban areas [125], geographic cancer distribution and prevalence is still a matter of investigation. Díaz et al. (2010) showed a higher prevalence of colorectal cancer in rural than in urban areas of Córdoba, Argentina [126]. Sharp et al. (2014), studying 20 types of cancers in Ireland, found that 12 of them were prevalent in urban areas [127]. However rural-urban area gradients are difficult to establish in Argentina since important socioeconomic diversity exists in both areas, and urban residence is not necessarily provided with better housing conditions, nutritional status, or health insurance availability [128]. 

Altogether, risky lifestyle behaviors were more prevalent in cases than in control subjects. This is corroborated by the combined analysis of seven lifestyle-related factors, which reveals a higher level of modifiable risky health behaviors in cases. Control subjects possessed about half of the unhealthy behaviors present in cases before diagnosis.

## 5. Conclusions

Our research provides data on Argentinean men that support recent trends worldwide on the potential influence of extrinsic risk factors on cancer, with insights into food habits and other lifestyle-related factors that may impact on the promotion of health or disease. To our knowledge, this is one of the first studies in South America concomitantly exploring food habits with several lifestyle-related factors of men with a cancer diagnosis. It was remarkable to observe the predisposition to positive changes in Argentinean men in the face of a cancer diagnosis, especially those associated with dietary aspects. This may be particularly linked with sociocultural characteristics of Latin Americans and further studies are warranted. Even though our study has a number of limitations, innate to observational approaches, it raises several questions and opens an avenue for future research, fostering investigations on cancer risk in men and providing cancer prevention strategies in South America.

## Figures and Tables

**Table 1:** Sociodemographic, environmental, and health characteristics of study participants.

Variables	Prostate Cancer (*n* = 326)		Other Cancers (*n* = 394)		Controls (*n* = 629)		*p* Value *
	*n*	%	*n*	%	*n*	%	
Age, mean (SD)	68.3 (8.2)		64.1 (10.4)		59.1(11.4)		<0.001
<50 years	10	3.1	39	9.9	155	24.6	
50–65 years	92	28.2	170	43.1	278	44.2	
>65 years	224	68.7	185	47	196	31.2	
Education							<0.001
Elementary school	171	52.5	141	35.8	249	39.6	
Middle and high school	104	31.9	151	38.3	219	34.8	
College	30	9.2	54	13.7	75	11.9	
University	21	6.4	48	12.2	86	13.7	
Marital status							<0.001
Singled	16	4.9	27	6.9	76	12.1	
Married	254	77.9	310	78.7	503	80	
Widowed	42	12.9	40	10.2	23	3.7	
Divorced	14	4.3	17	4.3	27	4.3	
Main labor activity							<0.001
Rural	88	27	81	20.6	167	26.6	
Industrial	44	13.5	58	14.7	97	15.4	
Commercial	95	29.1	91	23.1	137	21.8	
Autonomous professional	10	3.1	23	5.8	57	9.1	
Other	89	27.3	141	35.8	171	27.2	
BMI							<0.001
Underweight	1	0.3	4	1	1	0.2	
Normal	143	43.9	184	46.7	160	25.4	
Overweight	122	37.4	166	42.1	316	50.2	
Obese	60	18.4	40	10.2	152	24.2	
Reason for medical visits							<0.001
Check-up	233	71.5	126	32	-	-	
Symptoms	93	28.5	268	68	-	-	
History of family cancer	225	69	241	61.2	256	40.7	<0.001
Prostatitis	49	15	15	3.8	63	10	<0.001
Urinary tract infections	16	4.9	14	3.6	27	4.3	NS
Arterial hypertension	156	47.9	171	43.4	236	37.5	0.007
Diabetes	41	12.6	70	17.8	83	13.2	NS
Thyroid dysfunction	0	0	2	0.5	16	2.5	0.001
Depressive disorder	25	7.7	34	8.6	46	7.3	NS
Major emotional stress	207	63.5	225	57.1	137	28.1	<0.001
Antihypertensive use	152	46.6	171	43.4	230	36.6	0.006
Use of statins	37	11.3	70	17.8	124	19.7	0.005
Use of Acetylsalicylic acid	78	23.9	112	28.4	141	22.4	NS
Agrochemical exposure							0.005
None	201	61.7	251	63.7	449	71.4	
Handle/apply/sell	50	15.3	56	14.2	87	13.8	
Indirect	75	23	87	22.1	93	14.8	
Exposure (years)							0.013
<20	89	27.3	101	25.6	136	21.6	
20 or more	36	11	42	10.7	44	7	
Organochlorine exposure	68	20.9	64	16.2	114	18.1	NS
Organophosphorous exposure	111	34	134	34	158	25.1	0.002
Pyrethroids exposure	33	10.1	30	7.6	74	11.8	NS
Other chemicals	28	8.6	27	6.9	52	8.3	NS
Exposure with no protection	31	62	32	57.1	31	35.6	0.004
Housing							<0.001
Urban	155	47.5	202	51.3	402	63.9	
Rural	171	52.5	192	48.7	227	36.1	

* *p*-value for χ^2^ test; NS: non-significant (*p* ˃ 0.05).

**Table 2:** Dietary habits pre- and post-diagnosis of cancer.

Food Frequency Intake	Prostate Cancer (*n* = 326)				McNemar’s Test *	Other Cancers (*n* = 394)				McNemar’s Test **	Controls (*n* = 629)		*p* Value χ^2^ Test
	Pre-		Post		*p* Value	Pre-		Post		*p* Value	*n*	%	Pre- & Post
	n	%	n	%		n	%	n	%				
Meats					<0.001					<0.001			<0.001
<3 days/week	33	10.1	142	43.6		45	11.4	232	58.9		169	26.9	<0.001
≥3 days/week	293	89.9	184	56.4		349	88.6	162	41.1		460	73.1	
Fish					<0.001					<0.001			NS
<3 days/week	313	96.0	229	70.2		382	97	297	75.4		603	95.9	NS
≥3 days/week	13	4.0	97	29.8		12	3.0	97	24.6		26	4.1	
Dairy					NS					0.05			<0.001
<3 days/week	92	28.2	95	29.1		125	31.7	150	38.1		128	20.3	0.012
≥3 days/week	234	71.8	231	70.9		269	68.3	244	61.9		501	79.7	
Fruits					<0.001					<0.001			<0.001
<3 days/week	186	57.1	133	40.8		212	53.8	133	33.8		174	27.7	0.051
≥3 days/week	140	42.9	193	59.2		182	46.2	261	66.2		455	72.3	
Red fruits					<0.001					0.004			<0.001
<3 days/week	257	78.8	122	37.4		213	54.1	247	62.7		261	41.5	<0.001
≥3 days/week	69	21.2	204	62.6		181	45.9	147	37.3		368	58.5	
Green salads					0.03					0.001			<0.001
<3 days/week	163	50.0	141	43.3		148	37.6	188	47.8		218	34.7	NS
≥3 days/week	163	50.0	185	56.7		246	62.4	205	52.2		411	65.3	
Cruciferous vegetables					<0.001					<0.001			<0.001
<3 days/week	313	96.0	234	71.8		375	95.2	288	73.1		559	88.9	NS
≥3 days/week	13	4.0	92	28.2		19	4.8	106	26.9		70	11.1	
Legumes					0.001					0.001			<0.001
<3 days/week	233	95.9	217	89.3		291	93.3	275	88.1		449	88.4	NS
≥3 days/week	10	4.1	26	10.7		21	6.7	37	11.9		97	17.8	
Nuts					<0.001					<0.001			0.011
<3 days/week	302	92.6	222	68.1		369	93.7	297	75.4		557	88.6	0.030
≥3 days/week	24	7.4	104	31.9		25	6.3	97	24.6		72	11.4	
Seeds					NS					0.039			<0.001
<3 days/week	236	96.7	232	95.1		303	97.1	296	94.9		464	85.0	NS
≥3 days/week	8	3.3	12	4.9		9	2.9	16	5.1		82	15.0	
Whole grains					<0.001					0.052			<0.001
<3 days/week	199	81.6	221	90.6		273	87.5	257	82.4		342	62.6	0.006
≥3 days/week	45	18.4	23	9.4		39	12.5	55	17.6		204	37.4	
Fat-Rich foods					<0.001					<0.001			<0.001
<3 days/week	123	37.7	223	68.4		118	29.9	233	59.1		404	64.2	0.010
≥3 days/week	203	62.3	103	31.6		276	70.1	161	40.9		225	35.8	
Sugar-Rich foods					0.007					NS			NS
<3 days/week	117	35.9	152	46.6		156	39.6	153	38.8		241	38.3	0.035
≥3 days/week	209	64.1	174	53.4		238	60.4	241	61.2		388	61.7	
Black tea					0.002					<0.001			0.038
<3 days/week	244	74.8	212	65.0		318	80.7	238	60.4		513	81.7	NS
≥3 days/week	82	25.3	114	35.0		76	19.3	156	39.6		115	18.3	
Coffee					<0.001					<0.001			<0.001
<3 days/week	148	45.4	232	71.2		221	56.1	302	76.6		439	69.8	NS
≥3 days/week	178	54.6	94	28.8		173	43.9	92	23.4		190	30.2	
Mate					0.017					<0.001			NS
<3 days/week	71	21.8	96	29.4		90	22.8	196	49.7		144	22.9	<0.001
≥3 days/week	255	78.2	230	70.6		304	77.2	198	50.3		485	77.1	
Water													0.001
<2 liters/day	187	76.6	-	-	-	200	64.1	-	-	-	348	63.7	
≥2 liters/day	57	23.4	-	-	-	112	35.9	-	-	-	198	36.3	

* McNemar’s test for Prostate Cancer group; ** McNemar’s test for Other Cancers group; NS: non-significant (*p* ˃ 0.05).

**Table 3:** Sleep, physical activity, and other lifestyle characteristics pre- and post-diagnosis of cancer.

Lifestyle Habits	Prostate Cancer (*n* = 326)				McNemar *	Other Cancers (*n* = 394)				McNemar **	Controls (*n* = 629)		*p* Value for χ^2^ Test
	Pre-		Post		*p* Value	Pre-		Post		*p* Value	*n*	%	Pre-/Post
	n	%	n	%		n	%	n	%				
Sleep					<0.001					0.003			NS/NS
6–8 h	179	54.9	139	42.6		234	59.4	196	49		392	62.3	
<6 h or >8 h	147	45.1	187	57.4		160	40.6	201	51		237	37.7	
Difficulty falling asleep	78	23.9	64	19.6	NS	72	18.3	81	20.6	NS	105	16.7	0.024/NS
Sleep interruptions	138	42.3	143	43.9	NS	150	38.1	131	33.3	NS	189	30	<0.001/0.003
Medications for sleeping	103	31.6	127	39	0.042	93	23.6	176	44.7	<0.001	95	15.1	<0.001/NS
Physical activity	90	27.6	64	19.6	0.004	148	37.6	74	18.8	<0.001	346	55	<0.001/NS
Tobacco use	105	32.2	90	27.6	<0.001	155	39.3	93	23.6	<0.001	159	25.3	<0.001/NS
Alcohol consumption	271	83.1	180	55.2	<0.001	329	83.5	230	58.4	<0.001	480	76.3	<0.001/<0.001
Daily	153	46.9	66	20.2		150	38.1	30	7.6		174	27.7	
Occasional or never	173	53.1	250	79.8		244	61.9	364	92.4		455	72.3	
Antidepressant use	22	6.7	72	22.1	<0.001	33	8.4	103	26.1	<0.001	35	5.6	NS/NS

* McNemar’s test for Prostate Cancer group; ** McNemar’s test for Other Cancers group; NS: non-significant (*p* ˃ 0.05).

**Table 4:** Risk factors associated with food habits and other lifestyle aspects of study participants.

Variables	*n*	Prostate Cancer (*n* = 243)	Other Cancers (*n* = 312)
		OR (IC 95%)	OR (IC 95%)
Meat consumption ≥3 days/week	894	2.45 (1.32–4.58)	1.73 (1.04–2.87)
Dairy consumption ≥3 days/week	809	NS	0.53 (0.35–0.81)
Red fruits consumption ≥3 days/week	464	0.24 (0.14–0.40)	NS
Daily consumption of 3 fruits ≥3 days/week	573	0.38 (0.23–0.62)	0.45 (0.29–0.88)
Green salad consumption ≥3 days/week	602	0.47 (0.30–0.73)	NS
Cruciferous vegetables consumption ≥3 days/week	73	NS	0.27 (0.10–0.70)
Whole grains consumption ≥3 days/week	288	NS	0.35 (0.21–0.56)
Fat-rich foods consumption ≥3 days/week	606	3.52 (2.16–5.71)	5.18 (3.39–7.85)
Black tea consumption ≥3 days/week	219	4.44 (2.54–7.77)	2.58 (1.58–4.22)
Coffee consumption ≥3 days/week	471	2.38 (1.45–3.66)	NS
Physical activity ≥3 days/week	474	0.52 (0.32-0.84)	NS
Major emotional stress	354	8.59 (5.08–14.50)	7.77 (4.83–12.50)
Use of statins	205	0.32 (0.17–0.38)	NS
Urban housing	589	0.54 (0.35–0.85)	0.65 (0.44–0.95)
Tobacco use			
Active	220	NS	3.70 (2.16–6.33)
Passive	144	NS	NS
Past	368	NS	NS
No	369	Reference	Reference

NS: non-significant.

**Table 5:** Accumulated risk factors of study participants.

Group	0		1		2		3		4		5		6		7	
	*n*	%	*n*	%	*n*	%	*n*	%	*n*	%	*n*	%	*n*	%	*n*	%
Controls	16	2.5	87	13.8	144	22.9	180	28.6	114	18.1	58	9.2	28	4.5	2	0.3
Pre-diagnosis *																
Prostate Cancer	1	0.3	20	6.1	44	13.5	51	15.6	76	23.3	78	23.9	50	15.3	6	1.8
Other Cancers	4	1.0	22	5.6	45	11.4	79	20.1	115	29.2	88	22.3	35	8.9	6	1.5
Post-diagnosis **																
Prostate Cancer	0	0.0	5	2.5	27	13.3	64	31.5	70	34.5	27	13.3	8	3.9	2	1.0
Other Cancers	0	0.0	3	1.2	14	5.6	52	21.0	103	41.5	60	24.2	14	5.6	2	0.8

The seven risk behaviors considered were: intake of fresh fruits <3 times/week; intake of red fruits <3 times/week; intake of green salad <3 times/week; intake of meats ≥3 times/week; intake of fatty foods ≥3 times/week; physical activity <3 times/week; current tobacco smoking. * *p* < 0.001 ** *p* = 0.001 for χ^2^ test.
